# PDE12 mediated pruning of the poly-A tail of mitochondrial DNA-encoded tRNAs is essential for survival

**DOI:** 10.1038/s44321-024-00171-6

**Published:** 2024-11-20

**Authors:** Chenxiao Yu, Marco Tigano, Erin L Seifert

**Affiliations:** MitoCare Center, Department of Pathology and Genomic Medicine, Thomas Jefferson University, Philadelphia, PA 19107 USA

**Keywords:** Genetics, Gene Therapy & Genetic Disease, Organelles

## Abstract

E Seifert, M Tigano, and C Yu discuss the study from Haute et al, published in this issue of *EMBO Mol Med*, that reports the first pathogenic variants in the human PDE12 gene causing neonatal mitochondrial disease.

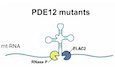

## Teaser

Mitochondrial DNA (mtDNA)-encoded RNA molecules undergo extensive processing to generate mature RNA, including removal of spurious poly-A tails by phosphodiesterase12 (PDE12). A new study by Van Haute and colleagues (Van Haute et al, 2024) describes the first pathogenic variants in the human PDE12 gene. The 3 missense mutations that were identified each carry severe phenotypic consequences that correlate with the presence or not of residual PDE12 protein, show cell-type-specific adaptive responses, and specificity in the mtDNA-encoded electron transport chain subunits that are most affected. These new data demonstrate the necessity of PDE12 for life, and provide invaluable insights into RNA processing in mitochondria.

**Figure 1:**
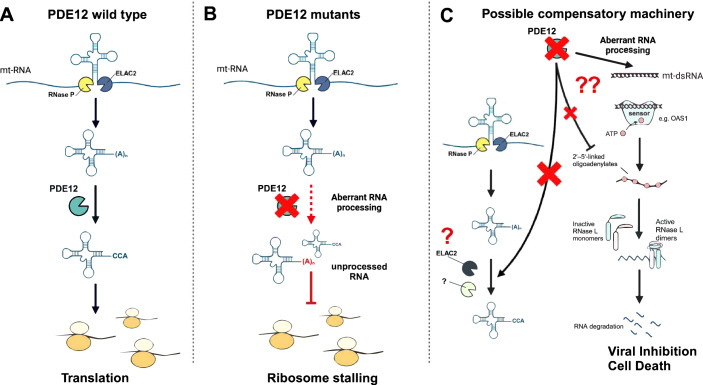
PDE12’s role in mitochondrial RNA processing and possible compensatory mechanisms in PDE12 pathological variants. (**A**) Schematic representation of PDE12’s role in mitochondrial RNA processing. PDE12 removes spurious poly-A tails from tRNAs and rRNAs to guarantee their performance in translation. (**B**) Schematic illustration of the consequences of PDE12 mutations. PDE12 pathological variants lead to aberrant mitochondrial RNA processing. Unprocessed mitochondrial RNA with spurious poly-A tails causes ribosome stalling, leading to decreased translation. (**C**) Adaptive and mal-adaptive mechanisms could be activated downstream of PDE12 mutations. (left) Even in the absence—or severe compromise—of PDE12 activity, cells can synthesize OXPHOS subunits, suggesting potential compensation for PDE12. A potential mechanism at play is the reliance on proteins capable of performing similar reactions. For example, ELAC2, typically involved in 3’ end processing of mitochondrial tRNAs, or other unknown protein(s), may partially compensate for PDE12 function by removing poly-A extensions from tRNAs and rRNAs. (right) PDE12 is a negative regulator of innate immunity. It is capable of processing 2’,5’-oligoadenylate (2–5 A), generated by OAS enzymes in response to dsRNA. In turn, 2–5 A activates RNase L, resulting in degradation of viral RNA and induction of apoptosis. In absence of PDE12, the aberrant processing of RNA could lead to the accumulation of immunogenic mt-dsRNA and the concomitant amplification of 2–5 A signaling cascades, with detrimental effects to the cell.

The mitochondrial genome (mtDNA) encodes 13 essential messenger RNAs (mRNA), 22 transfer RNAs (tRNAs), and 2 ribosomal RNAs (rRNAs), and these provide essential protein subunits of oxidative phosphorylation (OXPHOS) complexes I, III, and IV and the ATP synthase, as well as all tRNAs and rRNAs needed for translation of those 13 subunits. The remainder of the protein machinery needed for replication, transcription, and translation is encoded by nuclear DNA (nDNA), highlighting the requirement for mtDNA-nDNA interplay in the successful synthesis of the 13 mtDNA-encoded proteins. Here, we focus on the proteins responsible for the extensive processing that is needed to generate mature RNAs in the mitochondria. Mitochondrial transcription is performed by POLRMT and gives rise to genome-length polycistronic transcripts containing tRNAs, rRNAs and mRNAs that require extensive post-transcriptional maturation steps. Primary transcripts undergo cleavage at tRNA sites—the “tRNA punctuation model”—by RNase P and ELAC2 (RNAse Z), effectively releasing individual mRNAs, rRNAs, and tRNAs. One of the several processing events that mRNA molecules undergo before translation includes adding a poly-A tail, a step performed by MTPAP. Conversely, spurious poly-A tails need to be removed from tRNAs and rRNAs to guarantee their performance in translation. This step is performed by the mitochondrial phosphodiesterase PDE12 (Fig. 1A) (Pearce et al, 2017). Elegant in vitro studies have provided mechanistic details to the poly-A pruning reactions performed on mt-tRNAs and -rRNAs by PDE12. As expected, a complete knock-out in PDE12 from cells causes abnormal polyadenylation and mitochondrial ribosome stalling (Desai et al, 2020). Yet, proof that these mechanisms are vital to a complex organism has been lacking. A new study by Van Haute and colleagues describes the first pathogenic variants in the human PDE12 gene, along with their severe phenotypic consequences, and thereby provides the first demonstration that PDE12—and presumably the reaction that it catalyzes—is necessary for life (Van Haute et al, 2024).

Van Haute et al identified three homozygous missense variants in the PDE12 gene across three unrelated families: c.464 A > G (p.Tyr155Cys) and c.1115 G > A (p.Gly372Glu) in exon 1, and c.122 G > C (p.Arg41Pro) in the mitochondrial targeting sequence (MTS). In all cases, the amino acid that is changed is evolutionarily conserved. The p.Tyr155Cys and p.Gly372Glu variants led to decreased—but not absent—PDE12 protein levels. The p.Arg41Pro variant impaired the mitochondrial import of PDE12 and was associated with a complete absence of PDE12 protein. Notably, the p.Arg41Pro variant was not compatible with life. A sibling pair was identified to harbor the p.Tyr155Cys variant (one sibling did not live beyond early infancy), and the individual identified with the p.Gly372Glu mutation also did not live beyond early infancy. Using fibroblasts procured from either the p.Tyr155Cys or the p.Gly372Glu PDE12 variants and control individuals, the authors detected increased polyadenylation on several mt-tRNAs and also on the 16S rRNA (the other was not tested). This change was of a similar magnitude to that in HEK293 cells in which PDE12 was knocked out (HEK-KO). Similarly, extended poly-As were present on mt-tRNAs in a liver sample from a fetus harboring the p.Arg41Pro variant. Unexpectedly, however, the protein abundance of mt-DNA-encoded OXPHOS subunits was not decreased in fibroblasts harboring either the p.Tyr155Cys or the p.Gly372Glu PDE12 variants, and nor was the O_2_ consumption rate. This is in contrast with data obtained from HEK-KO cells in which the same subunits were decreased, presumably reflecting translation defects due to stalling of the mitoribosome. Skeletal muscle samples could be obtained from the only living sibling carrying the p.Tyr155Cys variant as well as from a p.Arg41Pro fetus. In this tissue type—and similar to HEK-KO cells—the protein abundance of mt-DNA-encoded OXPHOS subunits was lower in the p.Tyr155Cys carrier, or almost absent in the p.Arg41Pro one. These changes correlate with the protein levels of PDE12, that were very low or not detected, respectively. Interestingly, in both cases, Complex I and IV subunits were more affected than Complex III or ATP synthase subunits, and a similar tendency was evident in the HEK-KO cells. Finally, transcriptomics analysis of fibroblasts carrying the p.Tyr155Cys or p.Gly372Glu variants revealed alterations in several pathways, including pathways related to growth and tissue development, which, together with evidence supporting elevated glycolysis, indicates that the variant-carrying fibroblasts, despite their normal bioenergetics and OXPHOS subunit abundance, were impacted by the PDE12 variants.

The work of Van Haute et al undoubtedly establishes that PDE12 protein is required for survival in humans, but also challenges the field with new questions. The study reported a clear cell-type difference in the impact of PDE12 variants on protein expression levels of mt-DNA-encoded OXPHOS subunits, even within the same individual (see protein expression data from patient 2 in Fig. 6 of Van Haute et al). Cell-type differences could reflect, for example, compensatory mechanisms to replace the function of the missing protein or to support downstream processes. For example, it was reported that KO of ELAC2 leads to the accumulation of 3’-unprocessed mt-tRNA (Brzezniak et al, 2011), suggesting that ELAC2 can remove poly-A extensions (Fiedler et al, 2015) and thus be functionally redundant to PDE12 (Fig. 1B). A compensatory function of ELAC2—or a yet to be identified protein—in the context of PDE12 mutation could explain the normal levels of OXPHOS subunits in variant-expressing fibroblasts. At the same time, the elevated levels of poly-A extensions suggest that this compensation, although sufficient to support OXPHOS expression, is not complete. In this scenario, one possibility is that a small pool of transcripts can be deadenylated by the residual activity of PDE12—or by ELAC2—leading to the synthesis of sufficient mature protein to meet the low energetic needs of fibroblasts. But it is noteworthy that the transcriptomics analysis revealed pathways differences in variant-expressing fibroblasts; thus, the PDE12 variant fibroblasts had altered biology, i.e., were responding to the presence of the variant.

Another consideration is whether stress responses are activated by PDE12 variant cells. Human pathogenic variants in nuclear genes encoding for mitochondrial proteins, and many mouse models of primary mitochondrial disease, feature the activation of the integrated stress response, the mTORC1 pathway and/or the innate immune response (Hathazi et al, 2020). The latter seems most likely to occur when the affected protein impacts mt-nucleic acids (Marques et al, 2024). Whether PDE12 variants, or PDE12 KO models, result in stress response activation, and whether this is cell-type specific, remains to be determined. An attractive possibility relates to the additional role of PDE12 as a regulator of innate immunity. PDE12 was reported to degrade 2′,5′-oligoadenylate (2–5 A) (Wood et al, 2015), produced by OAS enzymes sensing immunogenic dsRNA. The 2–5 A can activate RNAse L leading to degradation of cellular (and viral) RNA and cell death, with the goal of minimizing viral spread (Hornung et al, 2014). In this way, absence of PDE12 could render cells more susceptible to the accumulation of unprocessed mitochondrial transcripts creating a basal pro-inflammatory state, while at the same time amplifying the 2–5 A signaling cascades, with detrimental effects to the cell (Fig. 1C). Whether mitochondrial dsRNA accumulates downstream of PDE12 deficiency, whether inflammatory responses are detected downstream of PDE12, and what role all these factors might play in the clinical variability of carriers, remain to be tested.

In conclusion, the discovery of pathogenic variants in PDE12 expands the list of nuclear-encoded mitochondrial proteins that cause primary mitochondrial diseases (PMDs). The screening of a wider population, including cases of unexplained fetal death or severe congenital abnormalities, will be instrumental to better understand the prevalence and spectrum of PDE12 mutations. Studies such as that by Van Haute et al—coupled with basic research—are critical in expanding our knowledge of PMDs, which have an all-cause (nDNA and mtDNA mutations) incidence of 1:4300 (Gorman et al, 2015). Despite this high incidence, PMDs are extremely heterogeneous in their presentation and therefore difficult to diagnose and manage, and are life-changing for the affected individual, family, and caregivers. The identification of new variants with bona-fide pathogenic consequences—such as those in PDE12—open the door to better and quicker diagnostic tools while also providing invaluable insights into the pathogenic mechanisms underlying mitochondrial dysfunction and the (mal)adaptive responses that different tissues adopt to cope. Patient-to-patient, or tissue-to-tissue variability in these (mal)adaptive responses could be the key to the extreme heterogeneity of PMDs presentation, one of the most challenging aspects of this disease class.

Graphics were created with BioRender.com.
